# Phospholipid and glycerolipid metabolism as potential diagnostic biomarkers for acute pancreatitis

**DOI:** 10.1186/s12944-024-02217-7

**Published:** 2024-07-23

**Authors:** Chunfeng Shi, Shengwei Liu, Meihua Zheng, Furong Yan, Dongyao Xu, Wei Wang, Jin Chen

**Affiliations:** 1 Department of Hepatobiliary and Pancreatic Surgery, the Second Affiliated Hospital of Fujian Medical University, Quanzhou, 362000 Fujian China; 2 Department of Hepatobiliary Surgery, the Second Affiliated Hospital of Xiamen Medical College, Xiamen, 36100 Fujian China; 3 Clinical Center for Molecular Diagnosis and Therapy, the Second Affiliated Hospital of Fujian Medical University, Quanzhou, 362000 Fujian China

**Keywords:** Acute biliary pancreatitis, Hyperlipidemic acute pancreatitis, Lipid metabolism, Biomarkers, Phospholipid, Glycerolipid

## Abstract

**Background:**

Acute pancreatitis (AP) is characterized as a systemic inflammatory condition posing challenges in diagnosis and prognosis assessment. Lipid metabolism abnormalities, especially triacylglycerol (TAG) levels, have been reported, indicating their potential as biomarkers in acute pancreatitis. However, the performance of the TAG cycle, including phospholipid and glycerolipid metabolism, in AP patients has not yet been reported.

**Methods:**

This study enrolled 91 patients with acute biliary pancreatitis (ABP), 27 with hyperlipidaemic acute pancreatitis (HLAP), and 58 healthy controls (HCs), and their plasma phospholipid and glycerolipid levels were analyzed through liquid chromatography‒mass spectrometry. The phospholipid and glycerolipid contents of plasma collected from AP patients on the first, third, and seventh days of hospitalization were also measured. An orthogonal partial least squares discriminant analysis model served to differentiate the ABP, HLAP and HC groups, and potentially diagnostic lipids were evaluated via receiver operating characteristic curves in both the test and validation sets. Correlations between clinical data and lipids were conducted using Spearman’s method. Clustering via the ‘mfuzz’ R package and the Kruskal‒Wallis H test were conducted to monitor changes during hospitalization.

**Results:**

Compared with those in HCs, the levels of phosphatidylcholine (PC), phosphatidylethanolamine (PE), and phosphatidic acid (PA) were lower in AP patients, whereas the levels of phosphatidylinositol (PI) and phosphatidylglycerol (PG) showed the opposite trend. Interestingly, TAG levels were positively correlated with white blood cell counts in ABP patients, and TAGs containing 44–55 carbon atoms were highly correlated with plasma TAG levels in HLAP patients. Phospholipid levels exhibited an inverse correlation with AP markers, in contrast to glycerolipids, which demonstrated a positive correlation with these markers. Additionally, PE (O-16:0/20:4) and PE (18:0/22:6) emerged as potential biomarkers because of their ability to distinguish ABP and HLAP patients from HCs, showing area under the curve (AUC) values of 0.932 and 0.962, respectively. PG (16:0/18:2), PG (16:0/20:4), PE (P-16:0/20:2), PE (P-18:2/18:2), PE (P-18:1/20:3), PE (P-18:1/20:4), PE (O-16:0/20:4), and TAG (56:6/FA18:0) were significantly changed in ABP patients who improved. For HLAP patients, PC (18:0/20:3), TAG (48:3/FA18:1), PE (P-18:0/16:0), and TAG (48:4/FA18:2) showed different trends in patients with improvement and deterioration, which might be used for prognosis.

**Conclusions:**

Phospholipids and glycerolipids were found to be potential biomarkers in acute pancreatitis, which offers new diagnostic and therapeutic insights into this disease.

**Supplementary Information:**

The online version contains supplementary material available at 10.1186/s12944-024-02217-7.

## Introduction

Acute pancreatitis (AP) ranks as a primary reason for hospitalization within the digestive system, with its incidence steadily increasing over the years due to the dietary habits and lifestyle changes [1]. The mortality ratio in severe acute pancreatitis (SAP) patients can rise to 30-40% [2]. Moreover, long-term sequelae, such as diabetes, necrotizing pancreatitis, recurrence and chronic pancreatitis, are common, resulting in an increasing financial burden [3]. Therefore, AP poses a threat to people’s health and financial security.

Gallstones and alcohol are the main contributors to AP, while hyperlipidemia ranks as the third most prevalent factor. Acute biliary pancreatitis (ABP) and hyperlipidaemic acute pancreatitis (HLAP) are frequent subtypes. Treatment depends on the cause. For ABP, early diagnosis is important because drainage of the biliary obstruction is urgently needed to avoid deterioration of the condition [4–6]. Although CT scans are crucial for diagnosis [7], obstruction of the bile duct caused by migratory gallstones and silt-like or transient stones is difficult to detect using CT scanning [8]. Unlike ABP, HLAP tends to present with a more severe condition and is more prone to progressing into SAP. CT scans serve as a significant diagnostic modality, but changes in CT images of necrotizing acute pancreatitis often occur 72 h after onset [6, 9]. Increased serum levels of amylase or lipase are among the criteria for AP diagnosis [7]. However, these levels are not directly linked to the severity or progression of AP. Furthermore, patients with HLAP may exhibit normal serum amylase and lipase levels [10]. Thus, identifying biomarkers for diagnosing and prognosing AP is essential.

Lipids play essential roles in cell metabolism, and their physiological and pathological functions have been a topic of interest in recent research. For example, polyunsaturated fatty acid metabolism is involved in inflammatory responses [11], and phosphatidylcholine metabolism contributes to resistance to cancer therapy [12]. Abnormal lipid metabolism in AP has been reported through metabolic profiling, and differential metabolism of phosphoric acid and glycerol has been described [13]. A Scottish epidemiological study revealed a direct link between plasma triglyceride levels and acute pancreatitis risk [14], and elevated triglyceride levels can predict increased severity and a greater probability of multiorgan disorder in AP patients [15–17]. That is, AP patients exhibit abnormal lipid changes in their plasma, particularly in the triglyceride cycle. However, research on plasma lipid metabolites in acute pancreatitis patients remains limited.

**Fig. 1:**
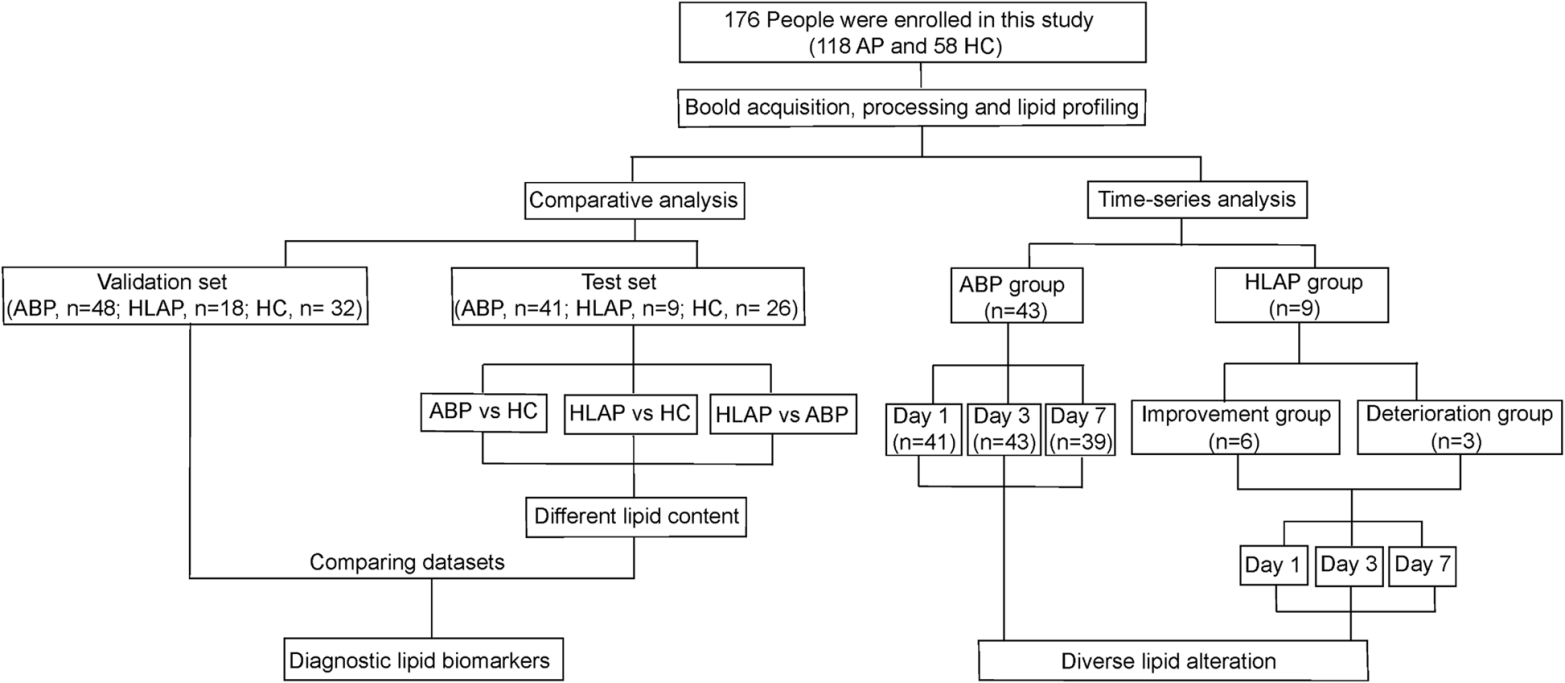
Flowchart of this study. AP, acute pancreatitis; HC, healthy control; ABP, acute biliary pancreatitis; HLAP, hyperlipidaemic acute pancreatitis. Day 1, first day of hospitalization; Day 3, third day of hospitalization; Day 7, seventh day of hospitalization

Phospholipid and glycerolipid metabolism involved in the triacylglycerol (TAG) cycle were profiled in patients suffering from ABP and HLAP, as well as in a control group of healthy individuals (Fig. 1), and potential biomarkers were identified. Furthermore, plasma was collected from ABP and HLAP patients at Days 1, 3 and 7 (D1, D3 and D7) of hospitalization, and lipid alterations were discussed in detail (Fig. 1). These findings reveal the molecular signatures of ABP and HLAP and provide potential prognostic biomarkers for AP. This study aimed to describe performance of the phospholipid and glycerolipid metabolism in ABP and HLAP patients and to identify potential biomarkers for AP diagnosis and prognosis.

## Materials and methods

### Study cohort

Between January 1, 2021, and December 31, 2022, a retrospective case‒control study involving 118 acute pancreatitis patients (AP group) and 58 healthy individuals (HC group) from the Second Affiliated Hospital of Fujian Medical University was conducted. The AP group includes patients meeting a minimum of two of the listed guidelines [18]: (1) upper abdominal pain; (2) CT, MRI, or abdominal ultrasound examinations indicate acute inflammatory changes in the pancreas; or (3) serum amylase or lipase levels increase to more than triple the normal upper limit. Specifically, in the absence of other aetiologies, ABP was diagnosed when imaging indicated biliary obstruction, and HLAP was diagnosed when plasma TAG levels exceeded 11.3 mmol/L [19]. Two patients in the ABP group were admitted 48 h after the onset of abdominal pain, and six patients improved and were discharged on the fifth day. Three patients in the HLAP group deteriorated and required the intensive care unit (ICU) admission, while six patients improved, and two of them were discharged on the sixth day. The HC group was not restricted by age or sex, and participants were eligible for inclusion if they met the following criteria: (1) no history of pancreatitis, hyperlipidaemia, biliary obstruction, liver or kidney dysfunction, rheumatic or immunological disorders, metabolic diseases, tumours, or excessive alcohol consumption; (2) normal physical examination results; and (3) no medication or recent infection. The AP group was subject to the following exclusion criteria: (1) received treatment within 72 h; (2) had acute or chronic pancreatitis, acute-on-chronic liver disease, or post-ERCP pancreatitis history; (3) were pregnant, lactating, or had end-stage liver or kidney disease, malignancies, metabolic disorders, or immunological diseases; and (4) had used immunosuppressive agents or steroids within the past three months. The study received ethical approval from the Ethics Committee of the Second Affiliated Hospital of Fujian Medical University (Ethics numbers: 2020-69 and 2023 − 470). All participants gave their informed consent and had the option to withdraw from the study at any point.

### Sample collection

During hospitalization, 2 mL of fasting venous blood was collected from AP patients on the first, third, and seventh days and from healthy volunteers on the day of their physical examination. Plasma was separated by centrifugation at 1800 rpm for 20 min and was subsequently stored at -80 °C. The entire sample collection process took less than 1 h. All the plasma samples used in this study were pooled together as quality controls (QCs) to evaluate the stability of the workflow.

### Lipid extraction

Lipid extraction was conducted as previously described [20]. Briefly, 20 µL of plasma was added to a mixture consisting of 225 µL of methanol (ANPEL Laboratory Technologies, Shanghai, China), 750 µL of MTBE (MACKLIN, Shanghai, China), and 188 µL of water (ANPEL Laboratory Technologies, Shanghai, China). This mixture was then centrifuged at 15,000 rpm for 15 min at 4 °C, resulting in three distinct layers: the lipid phase, aqueous phase, and solid residue. The upper 700 µL was collected and lyophilized for further liquid chromatography‒mass spectrometry (LC‒MS) analysis.

### LC‒MS analysis

LC-MS analysis was conducted following the previously established protocol [21]. In brief, a 4500 QTRAP system (AB SCIEX Pte. Ltd., Framingham, MA, USA) coupled to LC with an ACQUITY UPLC BEH HILIC column (100 mm × 2.1 mm, 1.7 μm, Waters) was used for separation. The lyophilized sample was dissolved in 100 µL of buffer with isopropanol (Merck, Darmstadt, Germany): acetonitrile (MACKLIN, Shanghai, China): water = 30:65:5 (v/v/v). One and 10 µL were injected for positive and negative mode analysis, respectively.

Lipid identification and quantification were conducted using SCIEX OS software. Multiple reaction monitoring in both positive and negative ion modes was employed to identify glycerolipids and phospholipids. Detailed information is provided in Supplementary Table S1.

### Statistical analysis

Lipids detected in 70% of the clinical samples were used for the subsequent analysis. The peak areas of the selected lipids were subsequently subjected to transformation and normalization. SIMCA (version 14.1) software was used for orthogonal partial least squares discriminant analysis (OPLS‒DA). The model’s effectiveness was assessed using cross-validation. Two-group comparisons were conducted using T tests and Mann‒Whitney U tests. The analysis of the study population’s baseline characteristics was analyzing by the chi-square test. For three-group comparisons, the Kruskal‒Wallis H test was used to evaluate changes. Differentially abundant lipids were identified using criteria of variable importance in projection (VIP) values > 1 and a false discovery rate (FDR) < 0.05. In this study, a *P* value < 0.05 was deemed to indicate statistical significance. The data underwent analysis using R (https://www.r-project.org/). Differentially abundant lipid graphs and receiver operating characteristic (ROC) curves were generated using GraphPad Prism 8.0.2. Clustering based on similar trends was performed using the ‘mfuzz’ package, and graphical representations were created using the ‘ggplot’ package in R.

## Results

### Study schematic

A total of 176 adults, including 118 patients with acute pancreatitis (male/female: 67/51) and 58 healthy controls (male/female: 30/28), were included in this study (Fig. 1). According to the different aetiologies, the 118 AP patients were categorized into 91 ABP patients (male/female: 47/44) and 27 HLAP patients (male/female: 19/8). Table 1 outlines participants’ characteristics, indicating similar distributions of sex and age among the three groups. Participant’s body mass index (BMI) of the HLAP group had a significantly upper when compared with the other two groups. The white blood cell (WBC) counts were greater in AP patients than in healthy individuals, likely because of inflammation. The study cohort was randomly divided into a test set (ABP/HLAP/HC: 43/9/26) and a validation set (ABP/HLAP/HC: 48/18/32), and the clinical characteristics of the two datasets are listed in Supplementary Tables S2 and S3, respectively. The test and validation sets presented similar clinical features to those of the full cohort.

**Table 1:** Clinical characteristics and laboratory indices of the study cohort

	HC group	ABP group			HLAP group			*P* value1	*P* value2	*P* value3
		D 1	D 3	D 7	D 1	D 3	D 7			
Patients (n)	58	89	43	37	27	9	7	-	-	-
Sex (male, %)	30 (51.7%)	46 (51.7%)	22 (51.2%)	17 (45.9%)	18 (66.7%)	8 (88.9%)	6 (85.7%)	0.292^a^	0.835^a^	0.315^a^
Age (years)										
median, IQR	41.5 (30.0–51.0)	48.0 (40.0–57.0)	48.0 (39.5–57.0)	49.0 (40.0–57.0)	41.0 (38.0–45.0)	40.0 (35.0–43.0)	42.0 (37.5–44.0)	0.070^b^	0.999^b^	0.458^b^
BMI (kg/m2)										
median, IQR	20.5 (18.0-23.9)	23.1 (19.7–24.9)	23.1 (19.8–24.6)	22.8 (19.7–24.6)	26.8 (26.5–27.8)	27.3 (26.7–27.8)	27.6 (26.7–27.9)	< 0.001^b^	0.956^b^	0.949^b^
WBC (x10^9/L)										
median, IQR	6.4 (5.2–7.5)	14.2 (11.1–16.3)	9.6 (7.8–12.8)	8.2 (6.2–10.9)	17.6 (13.5–21.1)	12.0 (9.1–18.9)	10.0 (4.8–16.6)	< 0.001^b^	< 0.001^b^	< 0.001^b^
AMS (U/L)										
median, IQR	-	1032.0 (541.0-1715.0)	219.0 (129.5-447.1)	98.0 (82.5–185)	496.5 (297.0-712.4)	219.0 (124.0-374.0)	124.3(80.0-208)	0.020^c^	< 0.001^b^	0.057^b^

Continuous variables are reported as medians with interquartile ranges (IQRs)

*P* value 1, comparison of healthy controls, ABP D1 and HLAP D1; *P* value 2, comparison of ABP D1, D3 and D7; *P* value 3, comparison of HLAP D1, D3 and D7

*HC* healthy control, *ABP* acute biliary pancreatitis, *HLAP* hyperlipidaemic acute pancreatitis, *D1, D3 and D7* diagnosis on the first, third, and seventh days of acute pancreatitis, respectively, *BMI* body mass index, *WBC* white blood cell count, *AMS* serum amylase

^a^Analysed by the chi-square test

^b^analysed by the ANOVA test

^c^analysed by the t test

To investigate dynamic lipid changes, AP patients on Days 1, 3, and 7 (D1, D3, and D7) of hospitalization were also included, and their characteristics are shown in Table 1; Fig. 1. In the comparison of the D1, D3, and D7 subgroups in the ABP group, both serum amylase (AMS) levels and WBC counts exhibited an overall decreasing trend with increasing length of hospitalization. These findings suggested that ABP patients were in remission. In the comparison of the D1, D3, and D7 subgroups in the HLAP group, three of them worsened during hospitalization. Sex, BMI, and AMS levels did not significantly differ at the various time points in HLAP patients.

Since TAG abnormalities have been observed in AP patients, a comprehensive description of the TAG cycle was investigated in this study. LC‒MS technology was used to detect glycerolipid and phospholipid levels in the ABP, HLAP and HC groups. Briefly, 29 positive-ion-mode features and 77 negative-ion-mode features were identified (Supplementary Table S1), including phosphatidylglycerol (PG), phosphatidic acid (PA), TAG, diacylglycerol (DAG), phosphatidylinositol (PI), phosphatidylcholine (PC) and phosphatidylethanolamine (PE).

To investigate the stability and reproducibility of the workflow, QC samples were analysed. Considering the relative standard deviation value, 100% and 90% of the lipids were less than 25% in positive and negative modes, respectively. Additionally, the Pearson correlation coefficient indicated a strong correlation (above 0.98) among QC samples in both ion modes (Supplementary Figure S1).

### Distribution of glycerolipids and phospholipids in the three groups

**Fig. 2:**
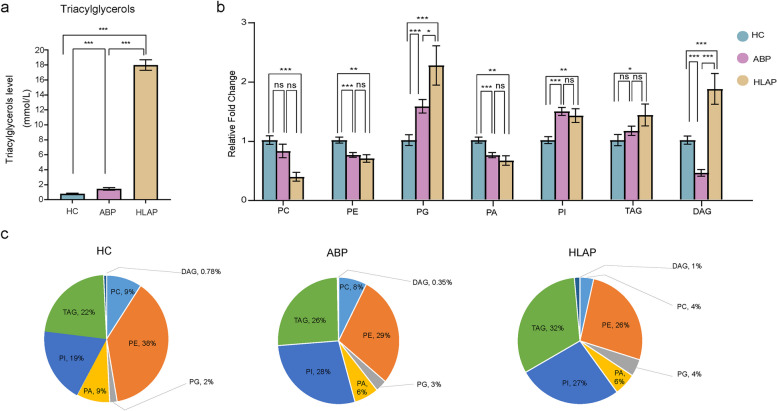
Distribution of glycerolipids and phospholipids in the three groups. **a** Expression of triacylglycerols detected in the clinic. **b** Relative abundance of lipid metabolites among the ABP, HLAP, and HC groups. **c** Proportion of lipid metabolites in the ABP, HLAP, and HC groups. HC, healthy control; ABP, acute biliary pancreatitis; HLAP, hyperlipidaemic acute pancreatitis. ns, none significant difference; *, *P* < 0.05; **, *P* < 0.01; ***, *P* < 0.001

According to the analysis of clinical biochemical markers, a significant increase in plasma TAG levels was observed in both ABP and HLAP patients compared with HCs (Fig. 2a). Increased TAG levels in patients with AP are consistent with previous observational study findings [16]. These findings indicate that the TAG cycle is abnormal in acute pancreatitis patients, and this study therefore focused on the expression of glycerolipids and phospholipids. Compared with healthy individuals, AP patients had lower levels of PC, PE and PA, whereas the opposite trend was observed for the levels of PG, PI, and TAG (Fig. 2b). In addition, TAG, PE, and PI constituted the major lipids among the three groups (Fig. 2c). Unsurprisingly, TAG was present at the highest level in the HLAP group (Fig. 2c). PE exhibited the highest level in the HC group, whereas PI levels were relatively high in all the AP patients (Fig. 2c).

### Differentially abundant glycerolipids and phospholipids in the three groups

**Fig. 3:**
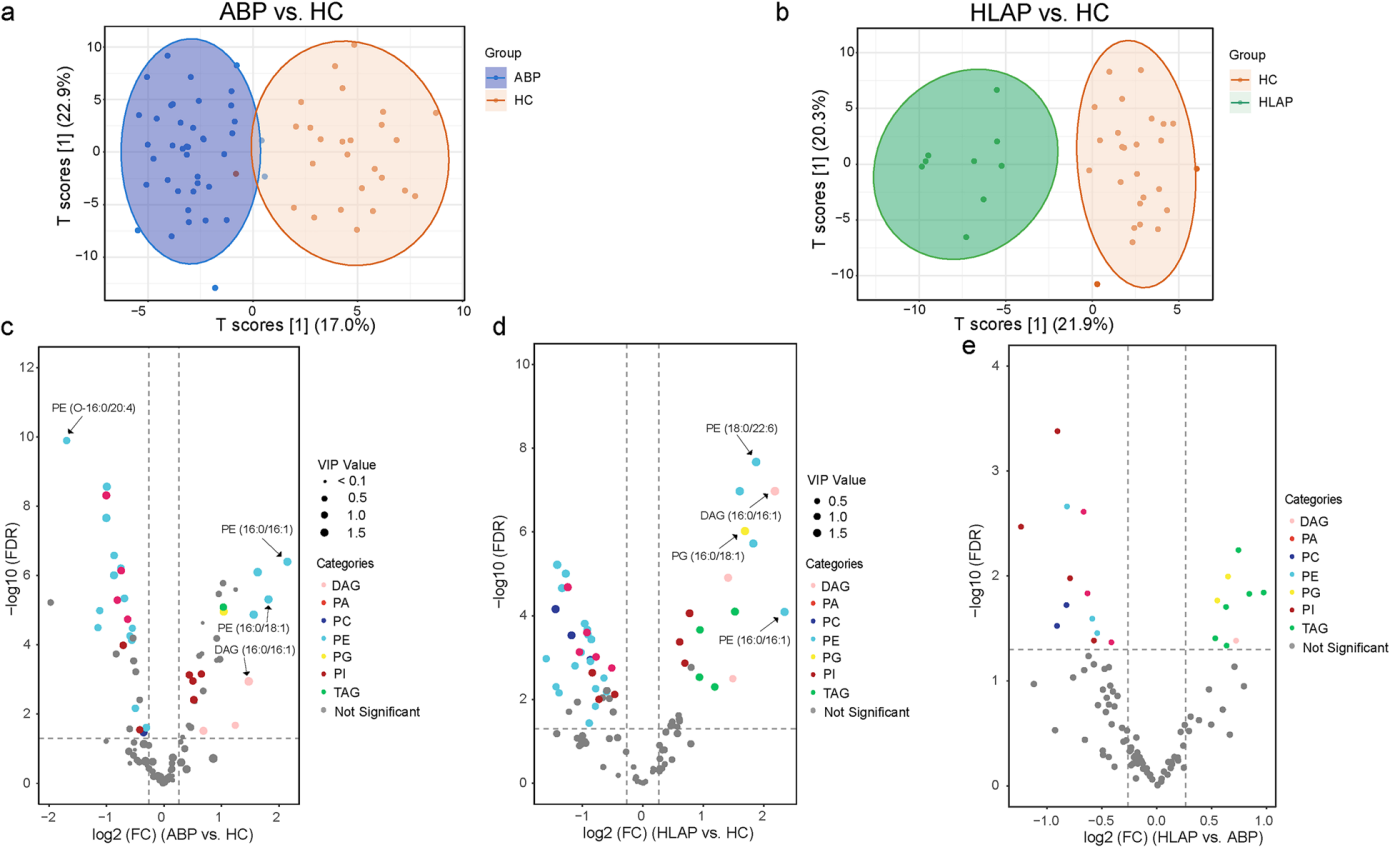
Differences in glycerolipid and phospholipid contents among the three groups. **a** and **b** OPLS-DA model between ABP and HC groups and between the HLAP and HC groups. **c**-**e** Volcano plots of ABP vs. HC, HLAP vs. HC, and HLAP vs. HC. Differentially abundant lipids were filtered according to the criteria of VIP > 1 and FDR < 0.05. In addition, thresholds of FC > 1.2 for upregulation and FC < 0.83 for downregulation were applied. OPLS-DA, orthogonal partial least squares discriminant analysis; VIP, variable importance in projection; FC, fold change; FDR, false discovery rate

Differentially abundant lipid metabolites among the ABP, HLAP, and HC groups, which could contribute to the early diagnosis of acute pancreatitis, were investigated. First, the OPLS-DA model was used to determine variations between groups (Fig. 3a and b). Remarkable separations were observed for both ABP vs. HC and HLAP vs. HC. Differentially abundant lipids were selected according to the criteria of a VIP values greater than 1 and a FDR less than 0.05. Additionally, a fold change (FC) threshold of more than 1.2 for upregulation and less than 0.83 for downregulation was applied. Compared with those in HCs, twenty-one lipids were downregulated and 13 were upregulated in ABP patients, among which PE was the main metabolite (Fig. 3c, Supplementary Table S4). Twenty-nine and 15 lipids were downregulated and upregulated, respectively, in HLAP patients compared with HCs (Fig. 3d, Supplementary Table S5). PE was also the main metabolite in this group. Interestingly, PE (16:0/16:1) exhibited the greatest increase in both ABP and HLAP patients, indicating its potential ability to distinguish AP patients from HCs (Fig. 3c and d). Notably, DAG (16:0/16:1) had the highest VIP score, indicating its potential role in differentiating AP patients from HCs (Fig. 3c and d). Although the HLAP and ABP groups could not be clearly separated (Supplementary Figure S3), twenty-one differentially abundant lipids were observed (Fig. 3e, Supplementary Table S6). Most of the upregulated lipids were TAGs, which is consistent with the characteristics of HLAP itself.

### Analysis of the clinical characteristics of the three groups

**Fig. 4:**
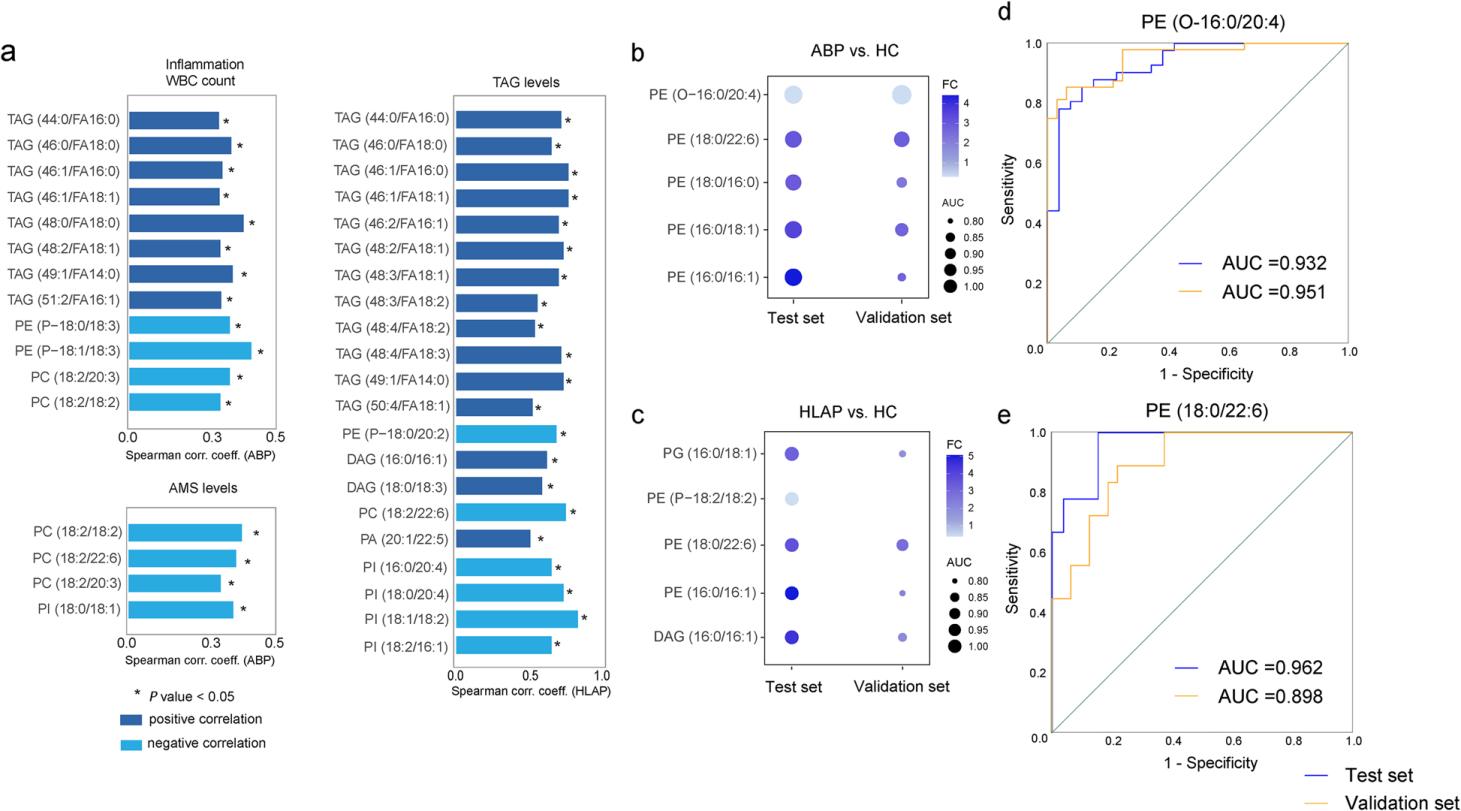
Clinical characteristics analysis and evaluation of the diagnostic performance of differentially abundant lipids in AP patients. **a** Spearman correlation analysis between clinical characteristics and lipids. **b**, **c** The top five lipids with good diagnostic performance in the ABP and HLAP groups in the test set, as well as their corresponding diagnostic performance in the validation set. The AUC values were obtained for comparisons of ABP patients vs. HCs and HLAP patients vs. HCs. **d**, **e** ROC curves for the test and validation sets of PE (O-16:0/20:4) and PE (18:0/22:6) in ABP patients vs. HCs and HLAP patients vs. HCs, respectively. WBC, white blood cell; AMS, serum amylase

To determine the clinical significance of lipids, their correlations with clinical characteristics, such as WBC count and AMS and TAG levels, were analysed via Spearman’s method (Fig. 4a). In the ABP group, TAG was positively correlated with the inflammatory marker WBC count. This finding suggests that these TAGs may be associated with inflammation. In the HLAP group, TAG expression was strongly correlated with plasma TAG levels, particularly with TAGs containing 44 to 50 carbon atoms. The lipids associated with AMS levels were mainly PCs, and they exhibited a negative correlation. Specifically, phospholipids were negatively correlated with AP disease markers, whereas glycerolipids were positively correlated with AP disease markers.

To investigate potential biomarkers, ROC curve analysis of differentially abundant glycerolipids and phospholipids was conducted (Supplementary Table S7). The lipids with the top five areas under the curve (AUCs) in the ABP or HLAP group are shown, which indicates their robust diagnostic performance (Fig. 4b and c). Ten potential biomarkers, eight of which are PEs, were identified. According to the ROC curves, PE (O-16:0/20:4) exhibited excellent diagnostic ability between the ABP and HC groups in both the test and validation sets, with AUCs of 0.932 (95% CI, 0.873–0.991) and 0.951 (95% CI, 0.909–0.993), respectively (Fig. 4d). Additionally, five lipids with the greatest AUC values in the test set can be used to distinguish HLAP patients from HCs, and their diagnostic ability was investigated in the validation set. PE (18:0/22:6) demonstrated strong diagnostic capability, achieving AUC values of 0.962 (95% CI, 0.904-1.000) in the test set and 0.898 (95% CI, 0.814–0.981) for the validation (Fig. 4e).

### Changes in glycerolipid and phospholipid contents during hospitalization in ABP patients

**Fig. 5:**
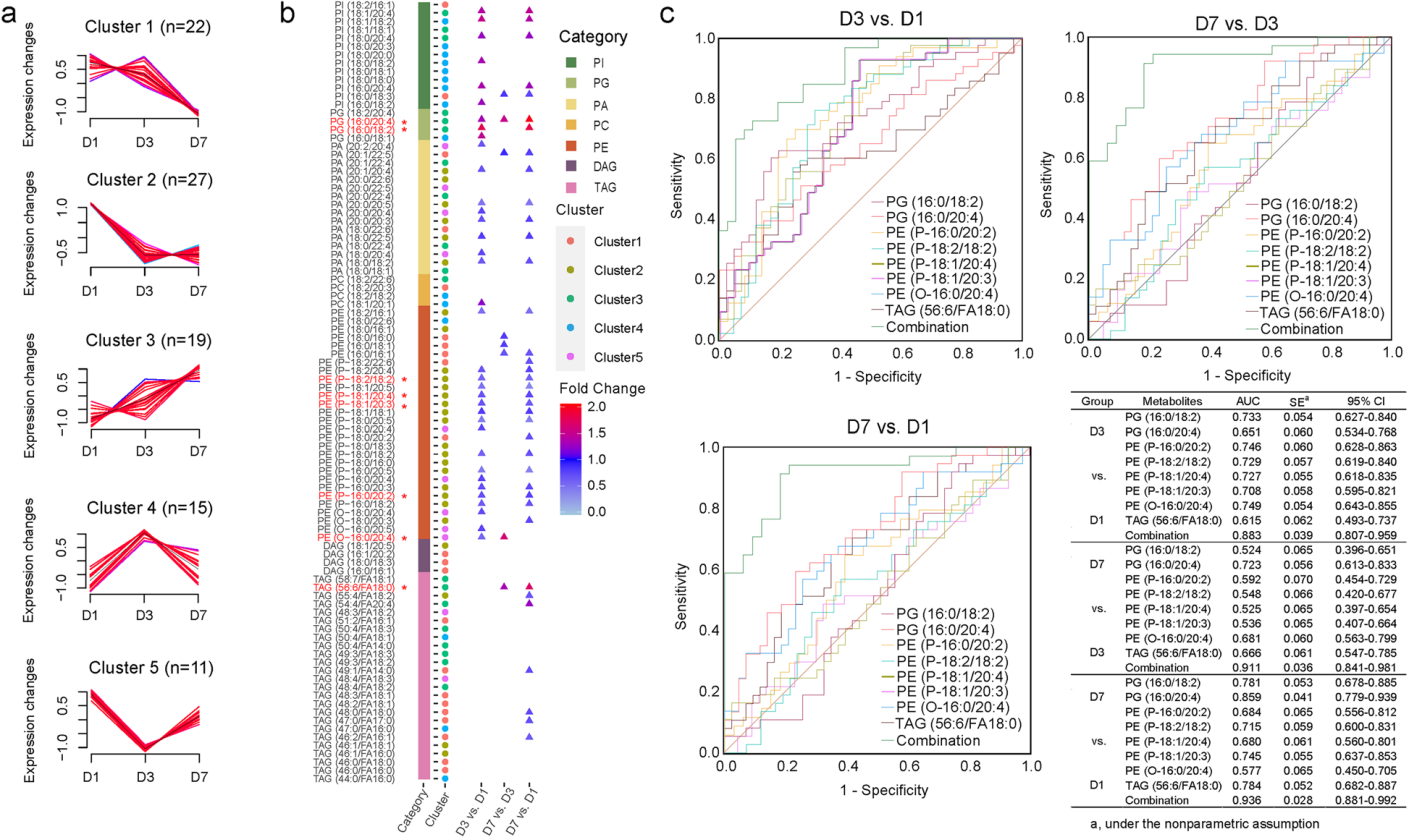
Dynamic changes in glycerolipid and phospholipid contents during hospitalization in ABP patients. **a** Time series analysis of glycerolipids and phospholipids. **b** Cluster attribution of lipids. Values highlighted in red and marked with “*” represent statistically significant differences in changes within the ABP group at D1, D3, and D7. **c** Evaluation of eight differentially abundant lipids during hospitalization. D1, 1st day of hospitalization; D3, 3rd day of hospitalization; D7, 7th day of hospitalization; *, *P* < 0.001

To profile the behaviours of the TAG cycle during hospitalization, blood was collected from ABP patients at Days 1, 3, and 7 (D1, D3, and D7) of hospitalization, and their lipid alterations were analysed in detail. Lipids were divided into 5 clusters according to their changes in the ABP group (Fig. 5a, Supplementary Table S8), and their detailed information is shown in Fig. 5b. In the ABP group, Clusters 1 and 2, which mainly contained PEs and TAGs, displayed overall decreasing trends. Most PEs were enriched in Cluster 2. The lipids in Cluster 3 exhibited an upregulated pattern, whereas Clusters 4 and 5 presented the highest and lowest expression, respectively, at D3. Eight lipids, PE (P-18:1/20:4), PG (16:0/20:4), PE (O-16:0/20:4), PG (16:0/18:2), PE (P-18:1/20:3), PE (P-16:0/20:2), TAG (56:6/FA18:0) and PE (P-18:2/18:2) significantly changed during the 7-day hospitalization (Kruskal-Wallis test, *P* < 0.001) (Fig. 5b). In addition, 36, 8, and 37 lipids were strongly altered between D3 and D1, between D7 and D3 and between D7 and D1 (Fig. 5b), respectively, according to the Mann‒Whitney U test. PE (O-16:0/20:4), which exhibited excellent diagnostic efficiency, also exhibited significant changes during ABP improvement. Notably, the lipids with marked differences were predominantly phospholipids, indicating their involvement in the onset and progression of ABP. The combination of these 8 lipids can be used to differentiate the stages of disease improvement. The AUCs were 0.883 (95% CI, 0.807–0.959), 0.911 (95% CI, 0.841–0.981) and 0.936 (95% CI, 0.881–0.992) for distinguishing D3 from D1, D7 from D3, and D7 from D1, respectively (Fig. 5c). PG (16:0/20:4) had good diagnostic ability for distinguishing ABP patients in the recovery phase from those in the acute phase, with an AUC of 0.859 (95% CI, 0.779–0.939) (Fig. 5c).

### Changes in glycerolipid and phospholipid contents in HLAP patients during hospitalization

**Fig. 6:**
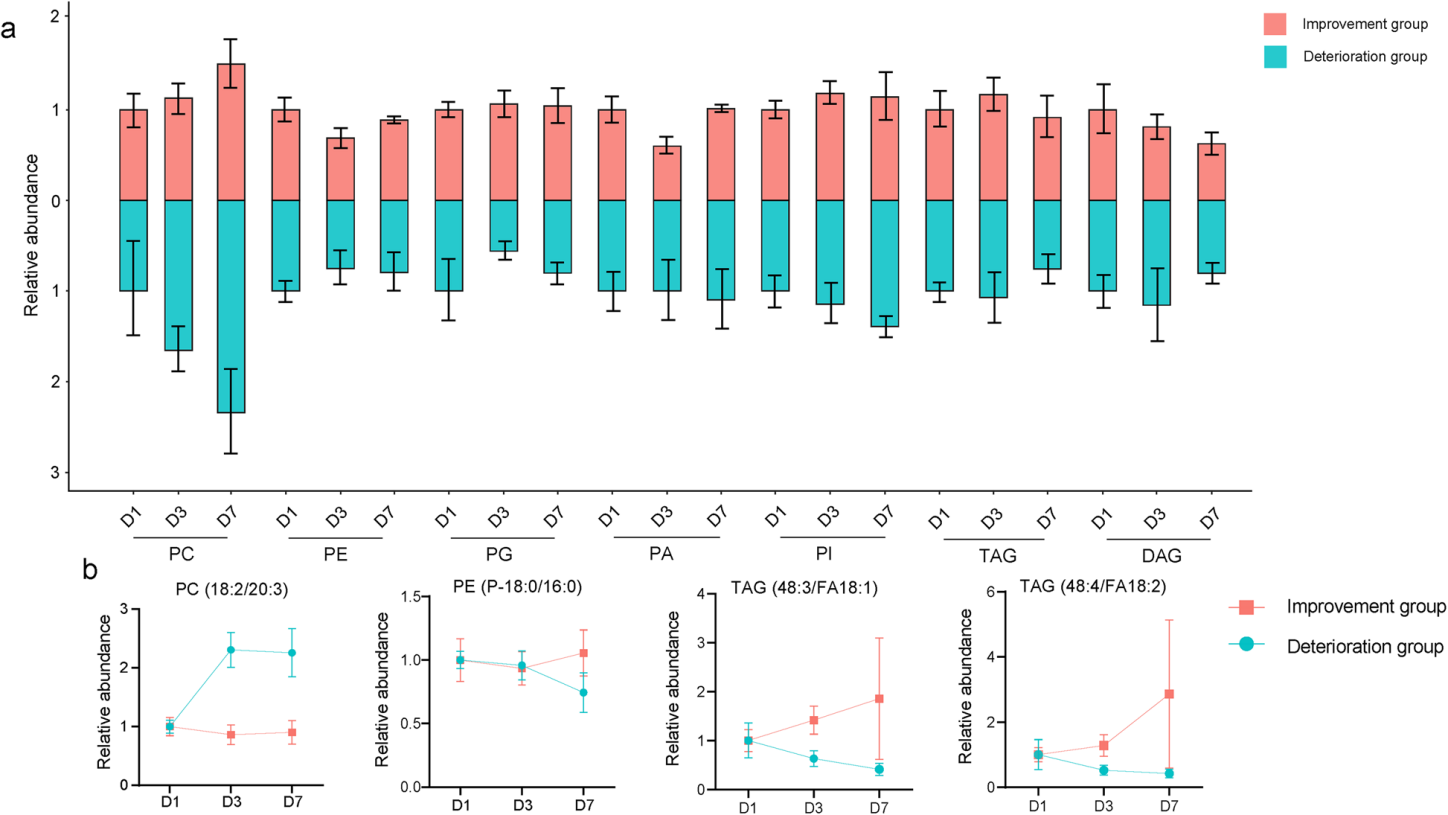
Dynamic changes in glycerolipid and phospholipid contents during hospitalization in HLAP patients. **a** Comparison of the relative abundances of glycerolipids and phospholipids at different time points during hospitalization between the improvement and deterioration groups. **b** Four lipids with opposite trends in the improvement and deterioration groups. *, *P* < 0.05

Three patients with HLAP deteriorated during hospitalization, and the remaining patients improved. To identify potential biomarkers to predict disease progression, the performance of glycerolipids and phospholipids was analysed. The changes in PG levels at different time points differed between the improvement group and the deterioration group (Fig. 6a). Although PC tended to increase in both groups, its fold change was more pronounced in the deterioration group than in the improvement group (Fig. 6a). Specifically, PC (18:0/20:3), TAG (48:3/FA18:1), TAG (48:4/FA18:2), and PE (P-18:0/16:0) tended to differ between the improvement and deterioration groups (Fig. 6b). The expression of PC (18:0/20:3) gradually decreased in the improvement group but showed the opposite trend in the deterioration group (Fig. 6b). PE (P-18:0/16:0), TAG (48:3/FA18:1), and TAG (48:4/FA18:2) all tended to decrease in the deterioration group (Fig. 6b). Notably, TAG (48:3/FA18:1) and TAG (48:4/FA18:2) not only displayed distinct trends during different disease stages but also exhibited a significant correlation with plasma TAG levels (Fig. 4a). Therefore, PE (P-18:0/16:0), TAG (48:3/FA18:1), and TAG (48:4/FA18:2) could be potential biomarkers for evaluating disease progression.

## Discussion

According to previous studies and the clinical data in this study, TAG was elevated in AP patients, including ABP and HLAP patients [16]. Therefore, this study focused on the metabolic abnormalities of glycerolipids and phospholipids involved in the TAG cycle, which have not yet been reported. Using targeted LC‒MS analysis, plasma lipid metabolites in acute pancreatitis patients and healthy individuals were quantified. Distinct lipid patterns were observed in patients with ABP and HLAP, and their alterations during hospitalization were fully discussed. This study represents the first comprehensive exploration of metabolic abnormalities in glycerolipids and phospholipids in patients with ABP and HLAP, offering novel insights and directions for clinical diagnosis and prognosis of AP.

Currently, diagnosing AP relies mainly on clinical indicators, with new discoveries emerging in recent years [22]. High TAG levels were identified as a contributing factor to the onset of AP [23]. However, the specific types of TAGs that have a significant impact on this disease remain unclear. A recent result from an animal experiment on SAP patients revealed that TAGs with 46–51 carbon atoms were significantly downregulated in plasma at 24 h [24]. However, this study revealed a significant positive correlation between TAGs with 44–50 carbon atoms and plasma TAG levels. This correlation may be due to species differences. Furthermore, the TAG level was identified as a predictor of deterioration in AP [16, 17], and different changes in TAG (48:3/FA18:1) and TAG (48:4/FA18:2) were observed between the improvement and deterioration subgroups of the HLAP group. On the other hand, TAG metabolism has been shown to regulate inflammatory responses [25], and a strong correlation between TAG and the inflammatory mediator WBC count was discovered in ABP patients.

Phospholipids are essential for the formation of cellular membranes, the regulation of inflammatory responses, and diverse physiological functions, and abnormalities in their metabolism are often associated with disease [26–28]. A negative correlation between phospholipids and disease-related markers was observed. The lipids with excellent diagnostic performance in distinguishing AP patients from HCs were predominantly phospholipids. A marked decrease in plasma phospholipids was reported among AP patients [13]. PG and PI were upregulated in AP patients. PG, which functions as a protective factor, has anti-inflammatory properties [29]. The integral role of PI cycling in cellular biology is underscored, with intricate connections with the onset and progression of AP [30]. Phospholipids were the main differentially abundant lipids in the comparison of different hospitalization time points in ABP patients. Notably, most of these lipids were PEs, fundamental components of biological membranes. They also regulate acinar cell autophagy and macrophage function, thus contributing to the pathogenesis of AP [31]. For the HLAP group, PE (P-18:0/16:0) showed opposite trends in the improvement and deterioration groups. PE is a potential diagnostic biomarker in AP patients.

### Study strengths and limitations

The main advantages of this research can be summarized as follows. First, this study is the first to describe the molecular features of phospholipids and glycerolipids in ABP and HLAP patients, and their relationships with clinical characteristics are fully discussed. Second, differentially abundant phospholipids and glycerolipids were discovered that can distinguish ABP and HLAP patients from HCs and showed great diagnostic ability. Thirdly, dynamic changes in important lipids were detected, suggesting their potential as biomarkers for monitoring the progression of ABP and HLAP. However, this study is not immune to certain methodological constraints. First, the sample size, particularly within the HLAP group, was relatively modest, potentially engendering statistical biases. Second, due to the differences in population characteristics between East China and West China, the incidence of acute pancreatitis also varied. Therefore, this study is not representative of the general population with acute pancreatitis. Therefore, future investigations should be predicated on multicentre collaborations with substantial clinical cohorts.

## Conclusion

This study revealed metabolic abnormalities in plasma glycerolipids and phospholipids in AP patients, which has significant clinical relevance. On the one hand, lipids such as PE (O-16:0/20:4) and PE (18:0/22:6) emerged as potential biomarkers for distinguishing ABP and HLAP patients, respectively, from HCs. Potential biomarkers could help clinicians accurately identify acute pancreatitis, allowing timely interventions and treatments to effectively alleviate patient symptoms. On the other hand, dynamic changes in PE (P-18:1/20:4), PG (16:0/20:4), PE (O-16:0/20:4), PG (16:0/18:2), PE (P-18:1/20:3), PE (P-16:0/20:2), TAG (56:6/FA18:0) and PE (P-18:2/18:2) may assist in predicting ABP improvement, whereas variations in the levels of PC (18:2/20:3), PE (P-18:0/16:0), TAG (48:3/FA18:1) and TAG (48:4/FA18:2) may serve as predictors of HLAP progression. These potential biomarkers could contribute to personalized treatment strategies to avoid severe complications and improve the outcomes of AP patients. In summary, these findings reveal potential biomarkers for diagnosing and prognosing AP, which will improve clinical decision-making in managing acute pancreatitis and personalize patient care.

### Supplementary Information

Supplementary Material 1.

Supplementary Material 2.

## Data Availability

No datasets were generated or analysed during the current study.

## Abbreviations

OPLS-DA: Orthogonal partial least squares discriminant analysis
HC: Health control
LC-MS: Liquid chromatography-mass spectrometry
HLAP: Hyperlipidemic acute pancreatitis
AP: Acute pancreatitis
ABP: Acute biliary pancreatitis
VIP: Variable importance in projection
PI: Phosphatidylinositol
PG: Phosphatidylglycerol
TAG: Triacylglycerol
FC: Fold change
DAG: Diacylglycerol
PC: Phosphatidylcholine
AMS: Amylase
QC: Quality control
AUC: Area under the curve
BMI: Body mass index
PE: Phosphatidylethanolamine
PA: Phosphatidic acid
FDR: False discovery rate
ROC: Receiver operating characteristic
SAP: Severe acute pancreatitis
FDR: False discovery rate
