# Supplementary material for: Phospholipid and glycerolipid metabolism as potential diagnostic biomarkers for acute pancreatitis
Source: Lipids Health Dis. 2024 Jul 23;23:223. doi: 10.1186/s12944-024-02217-7 (PMC11265382; doi:10.1186/s12944-024-02217-7)
Supplement: Supplementary file 2 — Supplementary Material 2. [file 12944_2024_2217_MOESM2_ESM.docx]

**Supplemental information**

**Phospholipid and glycerolipid metabolism as potential diagnostic biomarker for acute pancreatitis**

Chunfeng Shi^1, #^, Shengwei Liu^1, 2, #^, Meihua Zheng^1^, Furong Yan^3^, Dongyao Xu^1^, Tong Zhang^1^, Wei Wang^1, *^, Jin Chen^3, *^

^1^Department of Hepatobiliary and Pancreatic Surgery, the Second Affiliated Hospital of Fujian Medical University, Quanzhou, Fujian, 362000, China.

^2^Department of Hepatobiliary Surgery, the Second Affiliated Hospital of Xiamen Medical College, Xiamen, Fujian, 36100, China.

^3^Clinical Center for Molecular Diagnosis and Therapy, the Second Affiliated Hospital of Fujian Medical University, Quanzhou, Fujian, 362000, China.

#Chunfeng Shi and Shengwei Liu contributed equally to this work.

*Corresponding author:

Jin Chen, chenjin@fjmu.edu.cn

Wei Wang, wangwei9909@fjmu.edu.cn

**Contents**

Figure S1. Stability assessment of QC sample.

Figure S2. OPLS-DA model of HLAP vs. ABP.

Table S1. The ion mode of lipid metabolites.

Table S2. Differential metabolites in the comparison between ABP and HC group.

Table S3. Differential lipid metabolites in the comparison of HLAP and HC group

Table S4. Differential lipid metabolites in the comparison of HLAP group and ABP group.

Table S5. The diagnostic significance of lipid metabolites among the ABP, HLAP, and HC groups.

Table S6. Different clusters of lipids in ABP group.


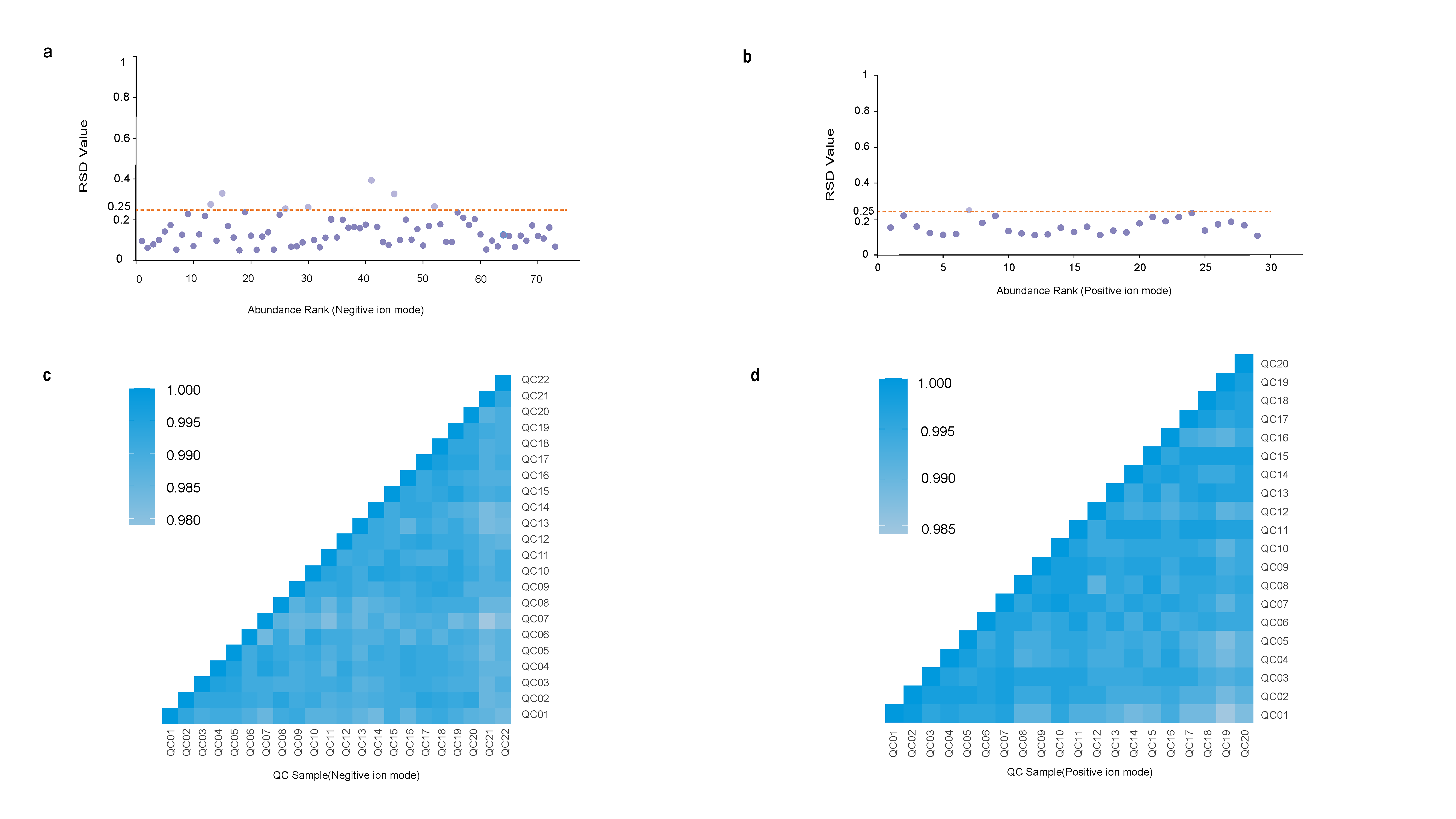


**Figure S1. Stability assessment of QC sample.** (a and b) Scatter plot of quality control samples in both negative and positive ion mode. The yellow dashed line on the y-axis in the graph represents an RSD equal to 0.25. (c and d) Correlation heatmap of QC samples in negative ion mode and in positive ion mode. RSD, relative standard deviation.


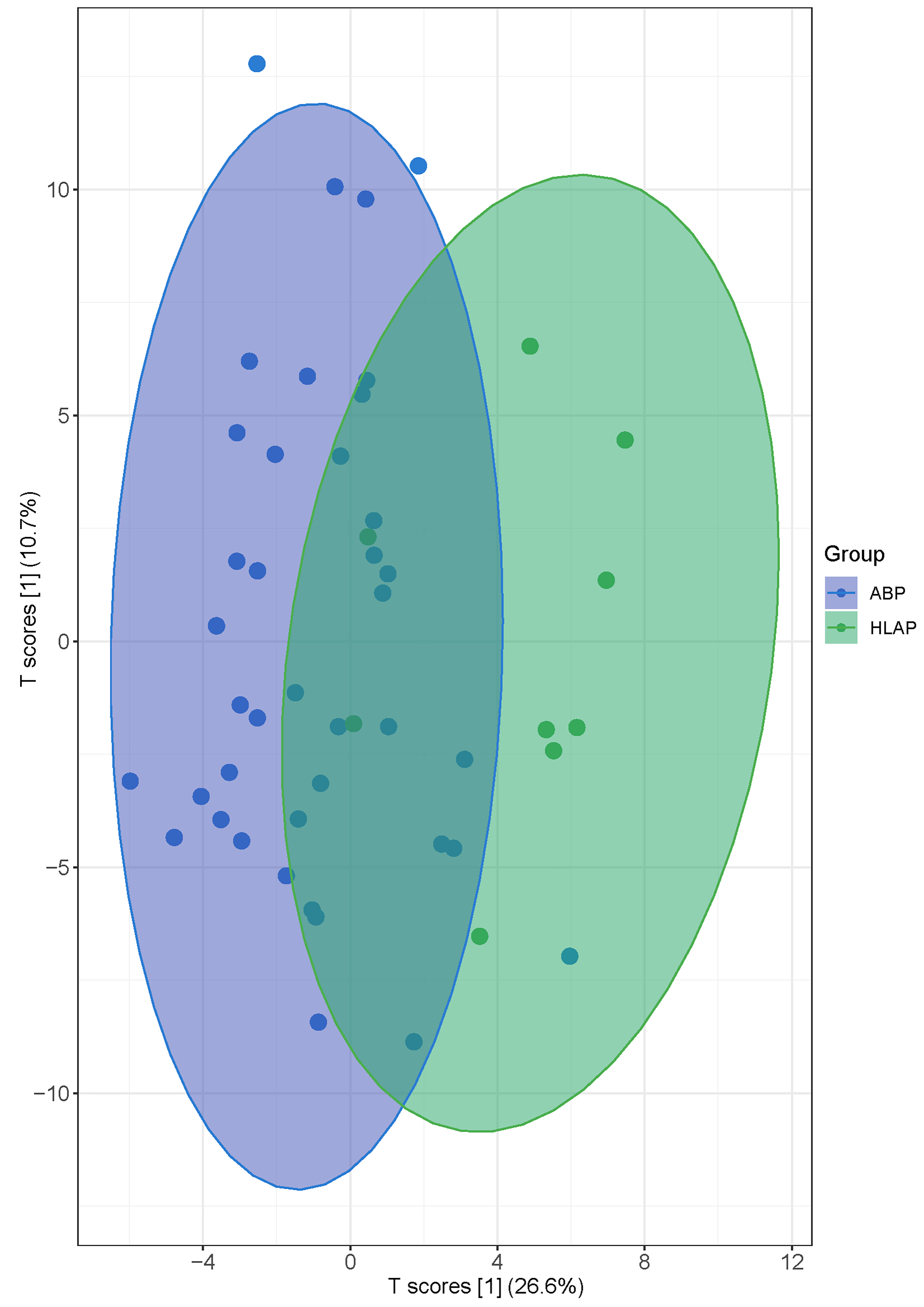


**Figure S2.** **OPLS-DA model of HLAP vs. ABP.**
